# Poly I:C causes exacerbation in a murine allergic inflammation model driven by house dust mite in Freund's complete adjuvant

**DOI:** 10.1186/1476-9255-10-S1-P21

**Published:** 2013-08-14

**Authors:** Jorge De Alba, Raquel Otal, Elena Calama, Félix Gil, Neil Gozzard, Montserrat Miralpeix

**Affiliations:** 1 Respiratory Therapeutic Area, Discovery, Almirall R&D Centre, 08980 Barcelona, Spain; 2 Immunology, UCB, Slough, Berkshire, SL1 3WE, UK

## Background

RNA viruses are major causes of respiratory infections and known to exacerbate asthma and other respiratory diseases. The objective of the study was to use poly I:C, a synthetic analogue dsRNA, to elicit exacerbation in a model of allergic inflammation driven by house dust mite (HDM) in Freund's Complete Adjuvant (FCA). This model is characterized by airway hyperresponsiveness (AHR) and a mixed T-helper phenotype [1].

## Materials and methods

BALB/c mice were sensitised subcutaneously on day 0 with HDM (100µg) in FCA as previously described [1]. On day 14, mice were exposed to saline or HDM (25µg) via intranasal instillation (i.n.). Poly I:C (30 µg) was administered i.n. 24hrs before (-24hr), at the same time (0hrs) or after (+6hours,+24hours) HDM challenge. 24 hours post-challenge, non-invasive whole-body plethysmography was used to assess AHR stimulated by aerosolised methacholine (MCh, 0-16mg/ml). 48 hours after HDM challenge, the bronchoalveolar lavage fluid (BALF) was collected to measure inflammatory cells.

## Results

Poly I:C exacerbated BALF neutrophils (-24, 0,+6), macrophages (-24, 0,+6) and lymphocytes (-24, 0) in the HDM challenged animals. At -24hrs or +6hrs, the AHR associated to MCh was also significantly exacerbated.

## Conclusions

Poly I:C exacerbates the inflammation and AHR in a murine model that mimics certain aspects of persistent asthma. This model could be used to investigate new mechanisms of action underlying viral exacerbation in persistent asthma and for the assessment and evaluation of novel therapies for such condition.
